# Effects of Heat Treatment on the Mechanical Properties and Thermal Stability of Bamboo

**DOI:** 10.3390/polym18172098

**Published:** 2026-08-29

**Authors:** Zilu Liang, Haiyun Jiang, Yimin Tan

**Affiliations:** School of Packaging and Materials Engineering, Hunan University of Technology, Zhuzhou 412007, China

**Keywords:** heat treatment, bamboo, mechanical properties, thermal stability

## Abstract

Bamboo contains abundant hydrophilic components such as hemicellulose which result in poor interfacial compatibility with epoxy resin and, consequently, limit its application in bamboo–epoxy composite packaging materials. In this study, we subjected bamboo (aged 3–4 years) to vacuum heat treatment to investigate the effects of treatment temperature (140, 160, and 180 °C) and holding time (4 and 6 h) and systematically evaluated the resulting changes in density, surface color, microstructure, mechanical behavior, and thermal stability. It was found that temperature serves as the dominant factor regulating bamboo color. With the increase in the heat treatment intensity, the lightness and yellowness of bamboo decrease, and the redness rises first and then falls, while the total color difference increases continuously. The optimal flexural strength and modulus of the treated bamboo are obtained at 140 °C, while its maximum tensile strength appears at 160 °C for 4 h. However, prolonged exposure at 180 °C causes marked mechanical degradation of the treated bamboo, which is attributed to the damaged fibrous structure. As for thermal stability, heat treatment removes heat-sensitive components, thereby increasing the 5% mass loss temperature and thermal degradation activation energy. Among all conditions, the sample treated at 160 °C for 6 h exhibits the best overall thermal stability, whereas excessive treatment at 180 °C destroys cellulose microcrystals and reduces the activation energy at high conversion rates. Considering the surface appearance, mechanical performance and thermal resistance comprehensively, the heat treatment at 140–160 °C with a 4 h holding time is the optimal modification process, which can provide theoretical and data support for the pretreatment of bamboo-based eco-friendly packaging composite materials.

## 1. Introduction

As a typical naturally renewable plant fiber, bamboo fiber features low density, high specific strength, easy availability of raw materials and superior environmental friendliness. It has emerged as one of the dominant reinforcing alternatives to synthetic fibers for green composite materials. Bamboo and bamboo-based composite materials have been used or investigated in engineering applications such as construction formwork and railway-car flooring [1,2]. Due to its advantages in biodegradability, structural versatility and environmental sustainability, it has gained great interest in the packaging field [3,4]. In detail, it can not only be fabricated into biodegradable bamboo-fiber cushioning packaging materials and eco-friendly packaging panels to replace traditional foams, plastics and wooden packaging products but also be processed into structural components for high-end gift packaging and load-bearing packaging assemblies for transportation, which satisfy multiple requirements, including shock absorption protection, structural support and environmental sustainability. Moreover, bamboo-derived materials have already been explored as biodegradable paper-based packaging tape and as bamboo-fiber-reinforced polymer packaging. In particular, bamboo fabric/poly(lactic acid) composites have been discussed for rigid and protective packaging, while bamboo-fiber-reinforced polymer composites have broader potential in packaging and transportation applications [5,6,7]. These reported applications support bamboo as a renewable packaging feedstock; however, the industrial scalability of the specific vacuum-treated bamboo strips investigated here remains to be established.

To further extend the applicability of bamboo fibers in high-performance packaging, they are often incorporated into polymer matrices to form composites. In addition to epoxy resin, relatively hydrophobic thermoplastic matrices such as polypropylene (PP) and poly(lactic acid) (PLA) have been investigated for bamboo-fiber composites [8]. Across these systems, the difference in surface polarity between hydrophilic bamboo and the polymer matrix can limit wetting and interfacial adhesion. Bamboo–epoxy composites combine the excellent moldability and corrosion resistance of epoxy resin with the reinforcing advantages of bamboo fibers, representing a vital research branch of natural fiber-reinforced composites. Nevertheless, their large-scale deployment in packaging and other industries is still restricted by insufficient material performance, which is primarily attributed to the poor interfacial compatibility between the hydrophilic bamboo reinforcement and the hydrophobic epoxy matrix. Bamboo contains abundant polar, hydroxyl-containing constituents, particularly cellulose and hemicellulose, which contribute to its hydrophilic nature. The relative contents of cellulose, hemicellulose, lignin, and extractives vary with bamboo species, culm age, and anatomical position, and, therefore, may influence its interfacial behavior with hydrophobic polymer matrices. Weak interfacial adhesion consequently occurs, thus deteriorating the mechanical properties and thermal stability of the composites. This critical bottleneck severely limits their extended utilization in high-end packaging structural components and temperature-resistant industrial packaging products [9,10,11,12,13].

Heat treatment serves as a green and efficient modification method for bamboo. Vacuum heat treatment removes moisture and volatile low-molecular-weight extractives from bamboo strips and promotes the release of volatile products such as acetic acid through partial hemicellulose degradation and deacetylation, thereby altering the chemical composition, microstructure, and apparent density of bamboo. During heat treatment, the loss of moisture and small molecular components is accompanied by thermal shrinkage and densification of cell walls, which increase the density of the bamboo strips and reduces their hydrophilicity simultaneously. These changes are expected to reduce the hydrophilicity mismatch between bamboo and hydrophobic polymer matrices, thereby providing favorable surface characteristics for subsequent composite fabrication [14,15,16]. However, excessively intense heat treatment not only impairs the surface appearance and texture of the bamboo strips and weakens their decorative performance [16] but also severely damages their internal microstructure, triggering deterioration in their mechanical properties [17], which further compromises the mechanical performances of bamboo–epoxy composites. Therefore, it is important to thoroughly understand how heat treatment affects the density, color, thermal stability, and mechanical properties of the bamboo strips, which is essential for both theoretical development and engineering applications.

Previous studies on thermally modified bamboo have predominantly focused on changes in surface chemistry, wettability, chemical composition, dimensional stability, or individual mechanical properties. Our previous work demonstrated that vacuum heat treatment effectively reduces hydrophilic groups and enhances the surface hydrophobicity of moso bamboo; however, whether these physicochemical improvements can be achieved without compromising the intrinsic load-bearing capability and thermal stability of bamboo remains unclear. Therefore, the present study systematically correlates heat-treatment severity with density, color evolution, microstructure, tensile and flexural properties, and thermal degradation behavior. In addition, the Flynn–Wall–Ozawa isoconversional method is employed to evaluate the evolution of thermal degradation activation energy. By integrating these complementary indicators, this work aims to establish the balance between beneficial thermal modification and heat-induced structural deterioration and to identify an appropriate pretreatment window for bamboo reinforcement in polymer composites.

## 2. Materials and Methods

### 2.1. Materials

Moso bamboo (3–4 years old, *Phyllostachys edulis*, harvested from Guangdong, China) was selected because this age range represents a mature stage commonly used for engineering applications. Bamboo chemical composition varies with species and culm age; therefore, bamboo of the same species and age range was used throughout this study to minimize the biological variability. According to previous studies on the same species, untreated moso bamboo typically contains approximately 46% α-cellulose, 24% hemicellulose, 26% lignin, 1–2% ash, and 2–3% extractives. First, the outer bast (phloem) and inner pith of bamboo culms were removed to acquire pure bamboo split substrates. Subsequently, the bamboo splits were processed into standard specimens with a thickness of approximately 1.7 mm, a width of 9.5 mm and a length of 30 mm through precision cutting. Then, the bamboo splits were heat-treated in a vacuum drying oven (DZF6020, Shanghai Yiheng Instruments Co., Ltd., Shanghai, China) under a relative pressure of −0.1 MPa at 140, 160, and 180 °C for holding durations of 4 and 6 h.

### 2.2. Density Measurement

Before density measurement, the bamboo strips (*n* = 3) were conditioned in a constant temperature and humidity chamber (HWS-150, Shanghai Jinghong Laboratory Instrument Co., Ltd., Shanghai, China; 0–75 °C, 35–95% RH) at 24 °C and 50% relative humidity until a stable mass was reached, thereby minimizing the influence of moisture-content variations among the specimens. The stable mass of each bamboo strip was weighed using an electronic balance. Because the precision-cut bamboo strips exhibited a regular rectangular geometry, their length, width, and thickness were measured using a vernier caliper (0–300 mm, Delixi Electric Co., Ltd., Yueqing, China) to calculate the geometric volume. The density of the bamboo strips was finally calculated using the formula ρ = m/V, where ρ is the density (kg·m^−3^), m is the constant mass of the conditioned specimen (kg), and V is the specimen volume (m^3^).

### 2.3. Measurement of the Color Difference

A 3nh NR60CP colorimeter (Shenzhen 3nh Technology Co., Ltd., Shenzhen, China) was used to detect the surface color variation of the bamboo strips (*n* = 3) before and after heat treatment. The CIELAB color space was adopted, where L* represents lightness (0 for black and 100 for white), a* denotes the red–green coordinate (positive for red, negative for green), and b* indicates the yellow–blue coordinate (positive for yellow, negative for blue). The total color difference, Δ*E*, of the bamboo strips under different heat treatment conditions was calculated based on the changes in L*, a* and b* to quantify the overall color variation under different treatment conditions. Especially, $\Delta E=\sqrt{{(\Delta L*}^{2}+{\Delta a*}^{2}+{\Delta b*}^{2})}$, where ΔL*, Δa*, and Δb* are the differences between the treated and untreated samples. The untreated bamboo was used as the reference. Two-way analysis of variance (ANOVA) was performed using Origin 2021 (OriginLab Corporation, Northampton, MA, USA).

### 2.4. Microstructure

The microstructural features of bamboo before and after thermal treatment were characterized using a scanning electron microscope (SEM, Apreo 2S HiVac, Thermo Fisher Scientific, Waltham, MA, USA).

### 2.5. Thermogravimetric Analysis (TGA)

The thermal stability of the bamboo strips before and after heat treatment was evaluated via thermogravimetric analysis (TGA 55, TA Instruments, New Castle, DE, USA). The testing temperature range was set from 30 to 600 °C at the heating rates of 5/10/15 °C min^−1^. The apparent activation energy was calculated using the Flynn–Wall–Ozawa isoconversional method over conversion levels of 10–50%. All tests were carried out under a nitrogen atmosphere.

### 2.6. Measurement of Mechanical Properties

The flexural properties of bamboo strips subjected to various heat treatment conditions were measured using a universal testing machine (CMT5105, MTS Systems (China) Co., Ltd., Shenzhen, China) in accordance with ASTM D790-25 standard [18], while their tensile properties were measured using an electronic universal testing machine (EM6.105, Shenzhen TestMate Instrument Equipment Co., Ltd., Shenzhen, China) following the ASTM D3039/D3039M-17(2025) standard [19]. Five samples for each group were tested.

## 3. Results and Discussion

### 3.1. Effects of Heat Treatment on the Density of the Bamboo Strips

**Figure 1:**
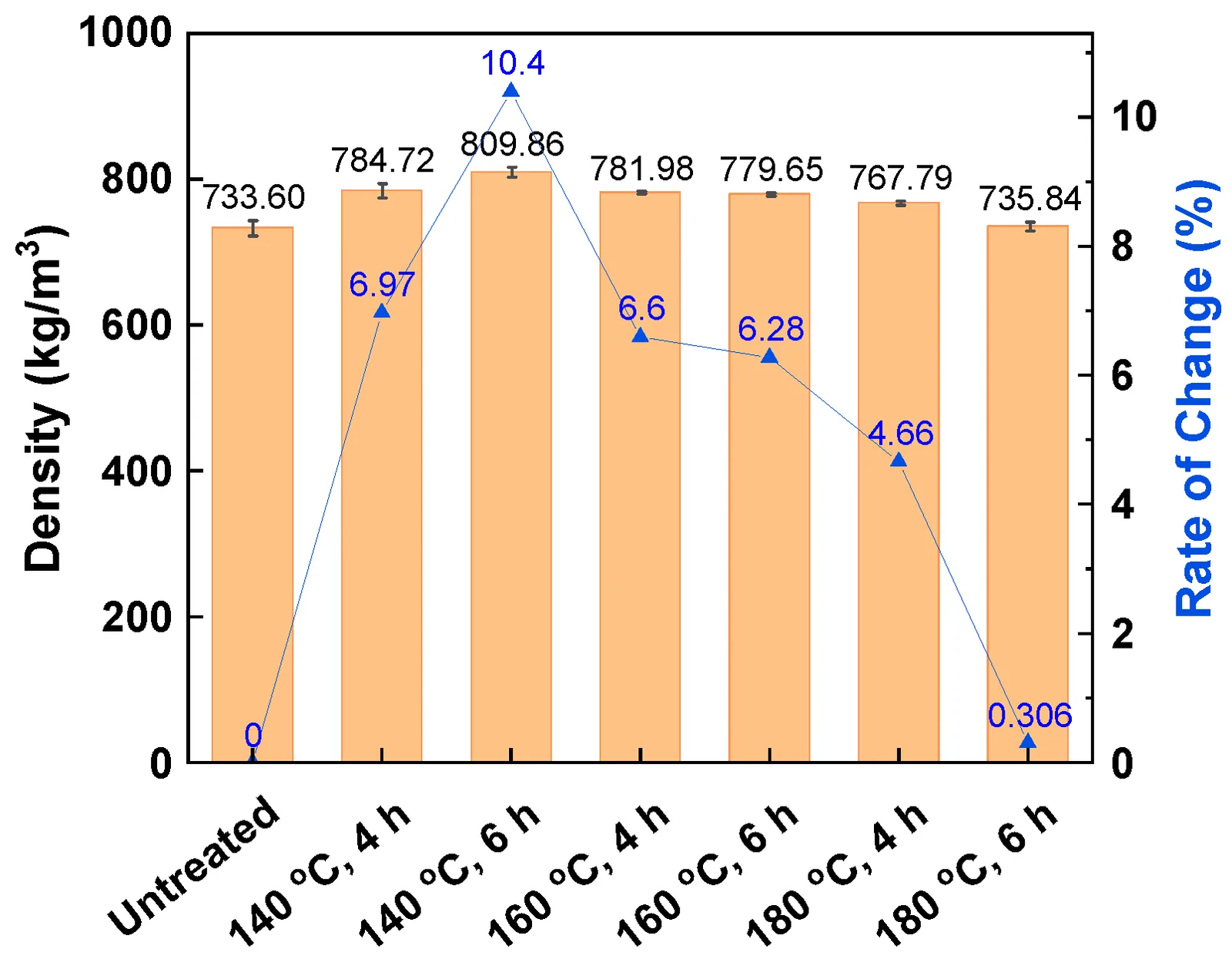
Effects of vacuum heat treatment on the density of the bamboo strips.

The density of bamboo strips is mainly determined by the content and compactness of the internal cellulose, hemicellulose and lignin. During heat treatment, the degradation and volatilization of these internal components trigger changes in the mass and volume of bamboo strips, thereby altering their density [20]. The density variations of the untreated bamboo strips and those treated under different conditions are presented in Figure 1.

The density variation of bamboo strips during heat treatment is governed by the following two competing effects: mass loss due to thermal degradation and dry shrinkage-induced densification of bamboo cell walls. Vacuum heat treatment removes internal moisture, extracts and hemicellulose, resulting in changes in density [21]. At identical heat durations, the density of the bamboo strips decreases continuously with rising temperature yet remains higher than that of the untreated samples.

Heat treatment at 140 °C increased the mean density of the bamboo strips, which rises continuously with an extended holding time and reaches a peak of 809.86 ± 6.74 kg·m^−3^ after 6 h of treatment at 140 °C, representing a 10.40% increase compared with untreated samples. At the low temperature of 140 °C, free water and low-molecular-weight extracts inside bamboo strips are removed. The loss of these hydrophilic components triggers substantial shrinkage of the bamboo cell walls and extensive closure of inherent pores, including native vessels and inter-fiber gaps, where a dry shrinkage-induced densification effect dominates the overall density change [22]. When the temperature rises to 160 °C, abundant hemicellulose undergoes thermal decomposition, generating numerous micropores within cell walls. The mass loss effect counteracts the densification, which greatly weakens the density-enhancement effect. Once the temperature increases to 180 °C, the hemicellulose is fully degraded, and the lignin suffers severe thermal cracking, resulting in a dramatic mass reduction of the bamboo strips. Meanwhile, thermal degradation generates massive microcracks and newly formed pores, accompanied by a remarkable rise in porosity and drastically aggravated density attenuation [23,24].

### 3.2. Effects of Heat Treatment on the Surface Color Difference of the Bamboo Strips

**Figure 2:**
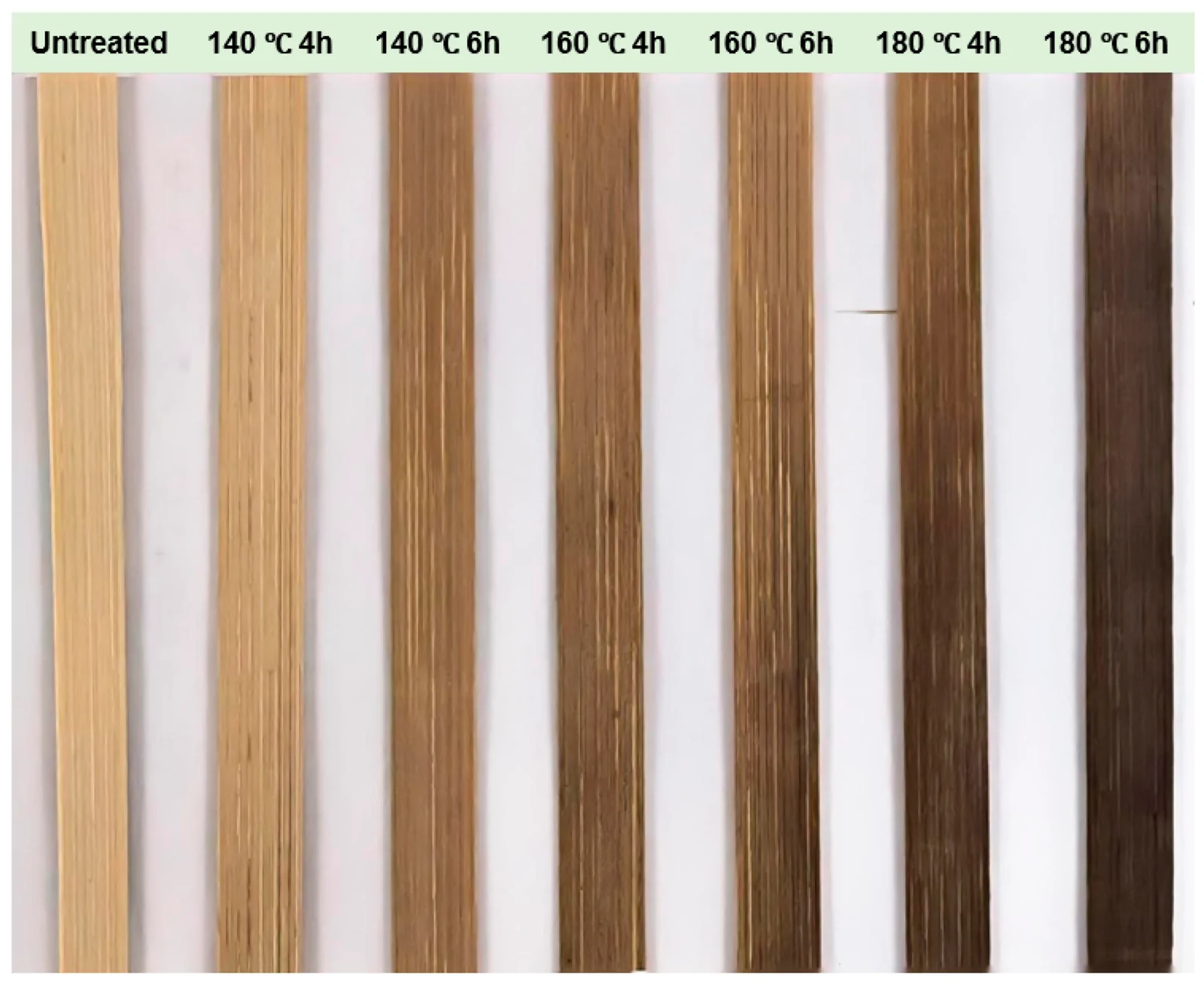
Photos of the bamboo strips treated under different conditions.

**Figure 3:**
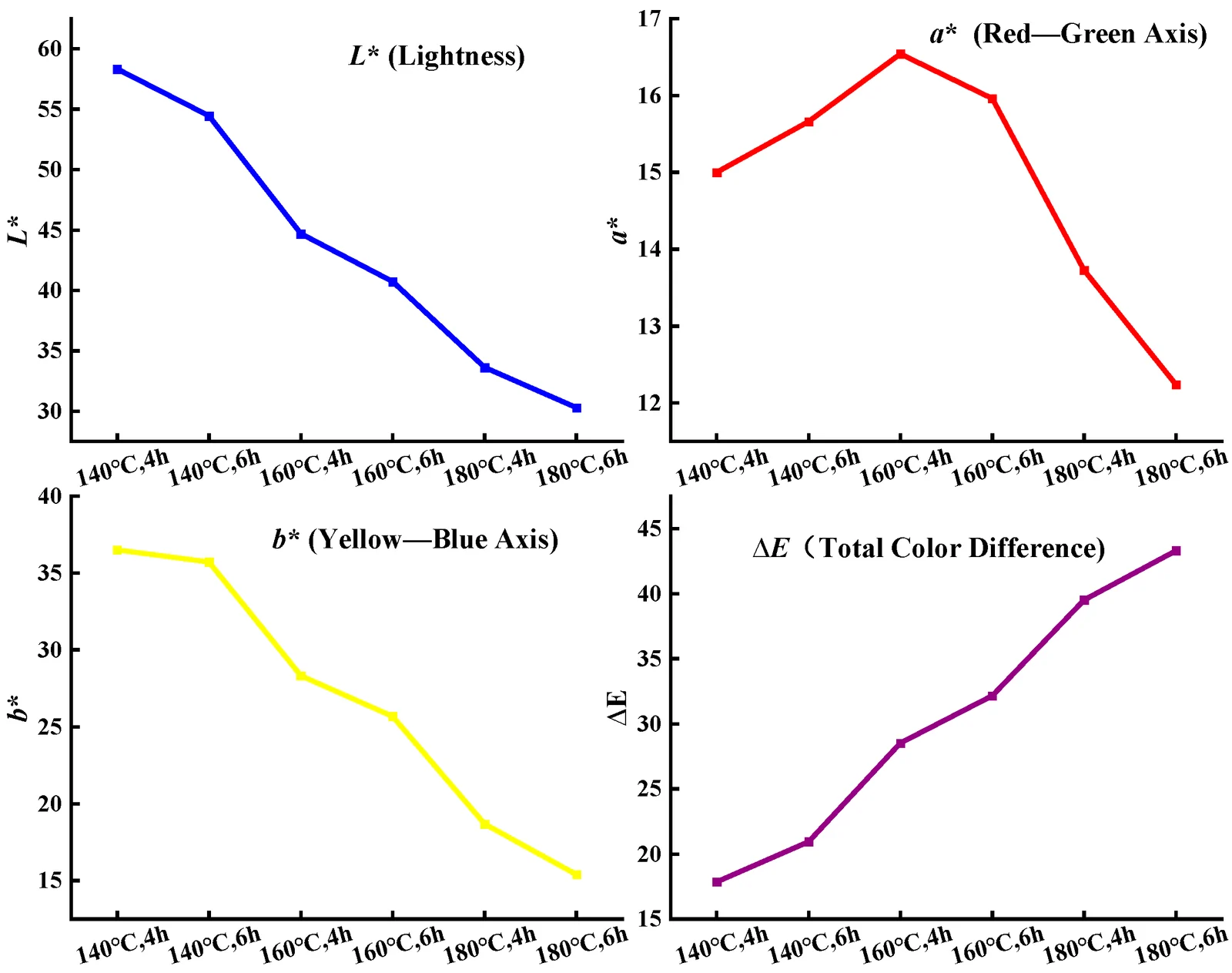
Variations in the color parameters (L*, a*, b*) and the total color difference (Δ*E*) of the bamboo under different heat treatment conditions.

Obvious surface color difference variations were observed on bamboo strips after heat treatment under diverse conditions, which are closely correlated with the thermal conversion of major chemical components and the microstructural evolution of bamboo [25]. The measured results are listed in Table 1. Heat treatment alters the chemical composition and microstructure of the bamboo strips, thereby changing their surface color, as indicated in Figure 2, and it can be found that temperature exerts a far more prominent effect than the holding duration. With the increase in the treatment intensity, the lightness (L*) and yellowness (b*) of the bamboo strips continuously decline, while the redness (a*) rises first and then decreases, accompanied by a persistent growth in the overall total color difference, Δ*E*, as illustrated in Figure 3.

L* represents the lightness of color; a higher value corresponds to brighter, whiter surfaces, while a lower value indicates darker, blacker surfaces. With an elevated heat treatment temperature and prolonged holding duration, the lightness, L*, of the bamboo strips gradually decrease from the initial state to 30.31, accompanied by continuous darkening of overall surface hue, as illustrated in Figure 3. For heat treatment at 160 °C, extending the holding time from 4 h to 6 h reduces the L* value from 44.68 to 40.73. In contrast, under identical holding time (4 h), raising the temperature from 160 °C to 180 °C lowers L* from 44.68 to 33.62, revealing that temperature exerts a stronger influence on L* than treatment duration.

Two plausible mechanisms account for this phenomenon. On the one hand, the hemicellulose inside the bamboo strips undergoes dehydration, deacetylation and chain scission degradation; meanwhile, the lignin experiences gradual thermal pyrolysis, condensation and carbonization, continuously promoting the formation of conjugated and other dark chromophoric structures associated with the thermal transformation of hemicellulose and lignin, as reported in previous studies. In addition, heat-induced microstructural changes may alter the surface light-scattering behavior and contribute to the apparent darkening of bamboo [24,26,27].

The a* value exhibits an increasing-then-decreasing trend and reaches a peak of 16.54 at 160 °C for 4 h. At moderate treatment temperatures, thermal transformation of lignin-related structures may promote the formation of red chromophoric species, contributing to the increase in a. With further increases in treatment severity, stronger optical absorption by dark thermally generated structures, together with further transformation of lignin-derived chromophores, likely suppresses the red component and leads to the subsequent decrease in a* [28].

With the increase in the heat treatment temperature and the extension of the holding time, the b* value of bamboo exhibits an overall trend of rising first and then declining. The maximum b* value decreases from 36.51 to 15.40, with a reduction of approximately 58%. This phenomenon can be explained as follows: under heat treatment at 140 °C, mild degradation of the hemicellulose and thermal transformation of the lignin generate chromophoric structures, resulting in an initial increase in yellowness. When the treatment temperature rises above 160 °C, the lignin begins thermal carbonization to form large-molecular brown and dark-brown structures, accompanied by continuous consumption of yellow substances and massive degradation of hemicellulose, which destroys the original yellow conjugated structures [24].

The Δ*E* monotonically rose with the increasing treatment intensity, growing gradually from 17.86 to 43.32. A two-way ANOVA showed that both temperature and holding time significantly affected the Δ*E*. Within the investigated ranges, temperature accounted for 95.23% of the total variation in Δ*E*, whereas the holding time accounted for 3.61%. The temperature × holding-time interaction was not significant and contributed only 0.03% of the total variation (Table S2). These results indicate that temperature was the predominant factor controlling the color change, although the smaller effect of holding time remained statistically significant.

### 3.3. Effects of Thermal Treatment on the Microstructure of the Bamboo Strips

**Figure 4:**
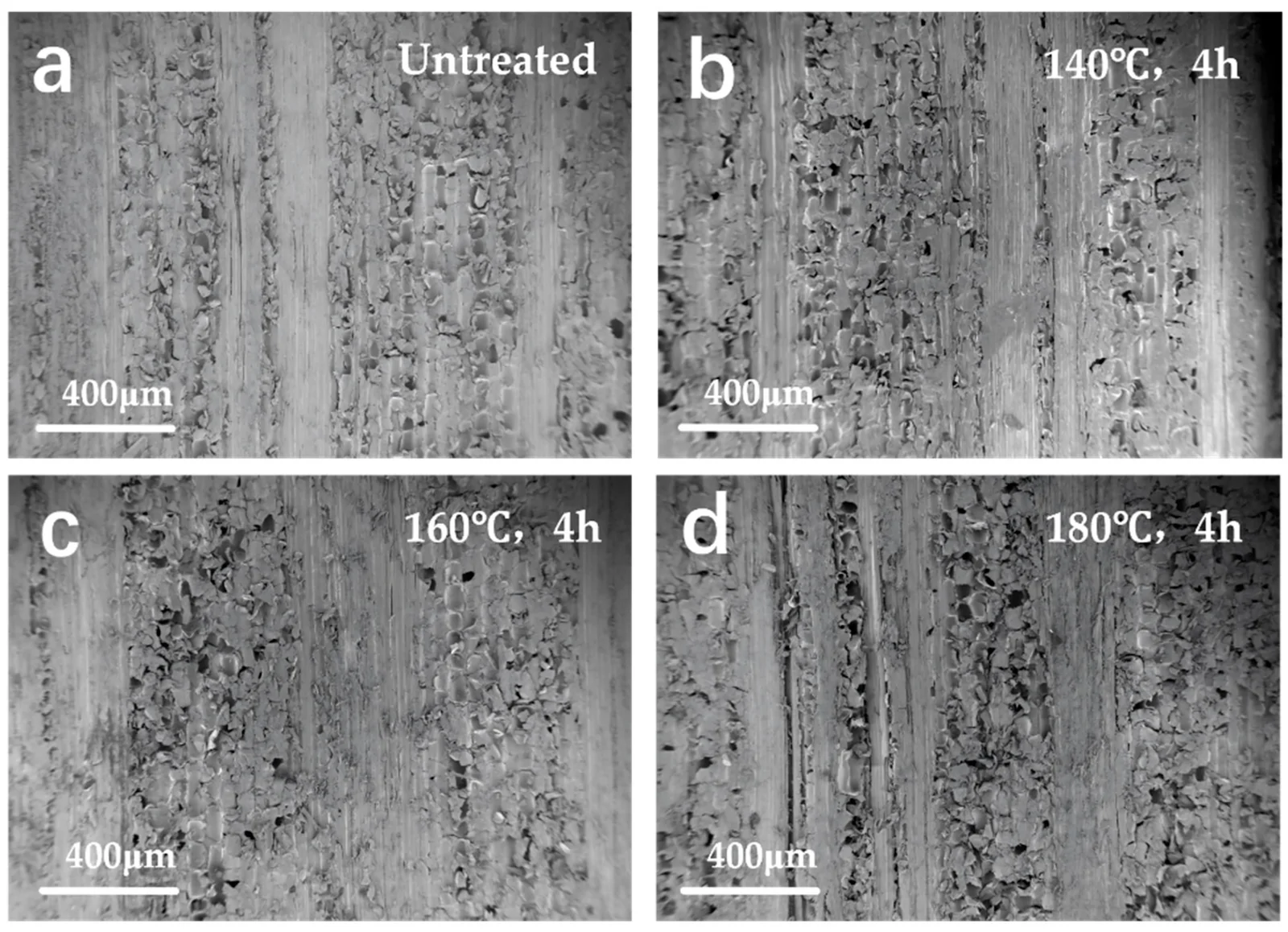
SEM images of the bamboo samples subjected to vacuum heat treatment at different temperatures: (**a**) untreated; (**b**) 140 °C for 4 h; (**c**) 160 °C for 4 h; (**d**) 180 °C for 4 h.

Thermal treatment alters the microstructure of bamboo and consequently changes its mechanical properties [29]. The SEM morphology of the untreated bamboo is presented in Figure 4a. The parenchyma cells are full and regularly arranged, the vessel walls are continuous and smooth, and only natural intercellular gaps exist. After thermal treatment at 140 °C for 4 h (Figure 4b), no obvious deterioration occurs. The fiber and vessel structures remain intact, with only a small number of tiny pores generated in the parenchyma tissue, which indicates that the main chemical components of bamboo do not undergo significant thermal degradation; only a small amount of moisture and extractives volatilize, causing negligible damage to the cellular skeleton. As the temperature rises to 160 °C (Figure 4c), the hemicellulose begins to decompose and leach out under heating. This greatly increases both the number and size of the pores in the parenchyma tissue and induces a slight collapse of some cells. Nevertheless, the main structures of the fiber bundles and vessels remain intact without irreversible damage, achieving controllable microstructural modification. When the temperature reaches 180 °C (Figure 4d), severe irreversible damage occurs. Massive thermal decomposition of the hemicellulose and lignin leads to the failure of the cell-wall supporting structures. Large-area interconnected pores form in the parenchyma tissue, accompanied by cell pulverization and collapse. Meanwhile, delamination and peeling appear on the vessel walls, and fiber bundles become loose, severely destroying the originally regular microstructure. The corresponding SEM images after 6 h of treatment are provided in Figure S1. Prolonging the treatment generally intensified the pore development and cell-wall damage, particularly at 160 and 180 °C.

### 3.4. Effects of Heat Treatment on the Mechanical Properties of the Bamboo Strips

Heat treatment at 140–180 °C modifies the surface chemistry and intrinsic structure of bamboo. Previous surface-characterization results indicate that the reduction in hydrophilic groups may favor compatibility with hydrophobic polymer matrices; however, the present study focuses specifically on the accompanying changes in the intrinsic mechanical properties of bamboo rather than on the performance of the final composite. Although heat treatment enhances the hydrophobicity of the bamboo strips, it degrades the hemicellulose and induces microdefects in the cell walls, thereby modifying tensile, flexural and other mechanical properties of the bamboo strips [16].

#### 3.4.1. Effects of Heat Treatment on the Tensile Properties of the Bamboo Strips

**Figure 5:**
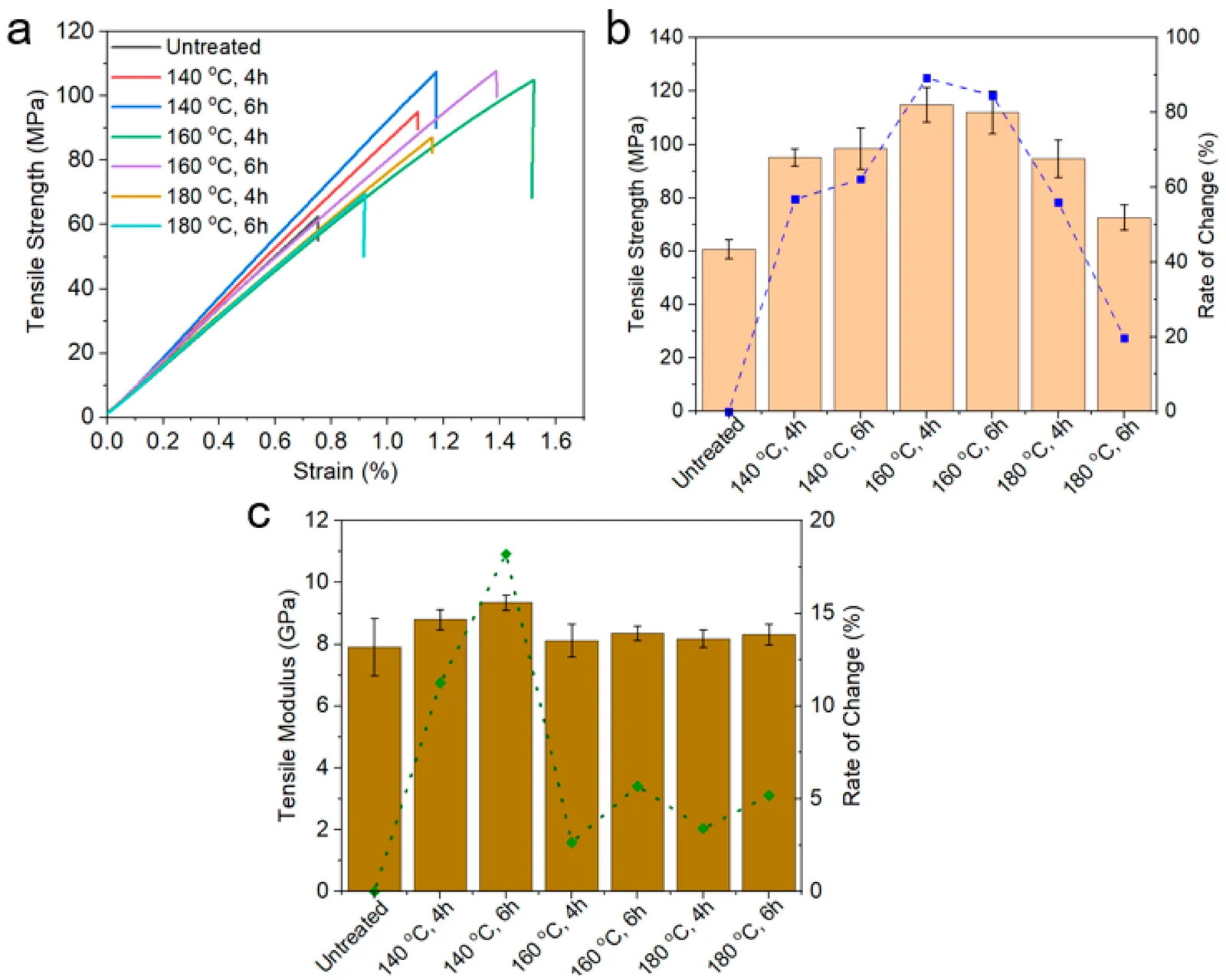
Effects of heat treatment on the tensile properties of the bamboo strips: (**a**) representative stress–strain curves; (**b**) tensile strength and its rate of change; (**c**) tensile modulus and its rate of change. Bars represent mean ± SD (n = 5), and the dashed lines with symbols represent the rate of change relative to the untreated bamboo.

The tensile properties of the bamboo strips are jointly determined by the cell-wall chemical components, intermolecular hydrogen-bond networks and interlayer interfacial bonding states. Heat treatment changes the contents of hemicellulose and lignin, as well as the fiber microstructure, which directly alters the tensile strength and elastic modulus of the bamboo strips. Different heat treatment temperatures and holding durations exert distinct effects on tensile properties [16]. The tensile properties of the bamboo strips before and after heat treatment are shown in Figure 5.

The tensile strength of the untreated raw samples is 60.71 MPa. At 140 °C, the tensile strength rises continuously with an extended holding time, reaching a 62.16% increment after 6 h of treatment. The tensile strength peaks at 160 °C for 4 h with an increment of 89.23%, and prolonged thermal exposure promotes further chemical degradation and the accumulation of microstructural defects, which together lead to a slight decrease in tensile strength. The reinforcing effect is drastically weakened at 180 °C, with only a 19.67% improvement in strength after 6 h of treatment.

The evolution of tensile strength does not follow the density variation directly because bulk density and axial load-bearing capacity are governed by different structural factors. At 140 °C, cell-wall shrinkage and structural densification dominate, producing the highest bulk density. When the treatment temperature increases to 160 °C, the partial degradation of hemicellulose causes additional mass loss and reduces density; however, the longitudinal cellulose-rich fiber framework remains largely intact. The preferential removal of the thermally less-stable hemicellulose phase, together with the increased relative contribution of cellulose and lignin, can maintain effective stress transfer along the fiber direction. Consequently, the tensile strength continues to increase and reaches its maximum at 160 °C for 4 h. With further increases in the treatment temperature or duration, degradation-induced micropores, microcracks, and damage to the fiber skeleton progressively outweigh these beneficial effects, leading to a decline in tensile strength [27].

The variation law of elastic modulus differs obviously from that of tensile strength. The elastic modulus of the untreated bamboo strips is 7.91 GPa. Heat treatment at 140 °C exerts a remarkable promoting effect on the modulus, with an increment of 18.20% after 6 h of treatment, the highest among all groups. The modulus increments of all samples treated at 160 °C and 180 °C are less than 6%.

At 140 °C, heat treatment may alter hydroxyl-associated intermolecular interactions and induce limited reorganization of lignin-related structures, which may contribute to the observed increase in stiffness [27]. Massive loss of hemicellulose at high temperatures generates micropores inside cell walls and weakens the interfacial bonding force between fibers, which counteracts the rigidity gain brought by lignin curing. As a result, the improvement range of stiffness is greatly restricted [9].

#### 3.4.2. Effects of Heat Treatment on the Flexural Properties of the Bamboo Strips

**Figure 6:**
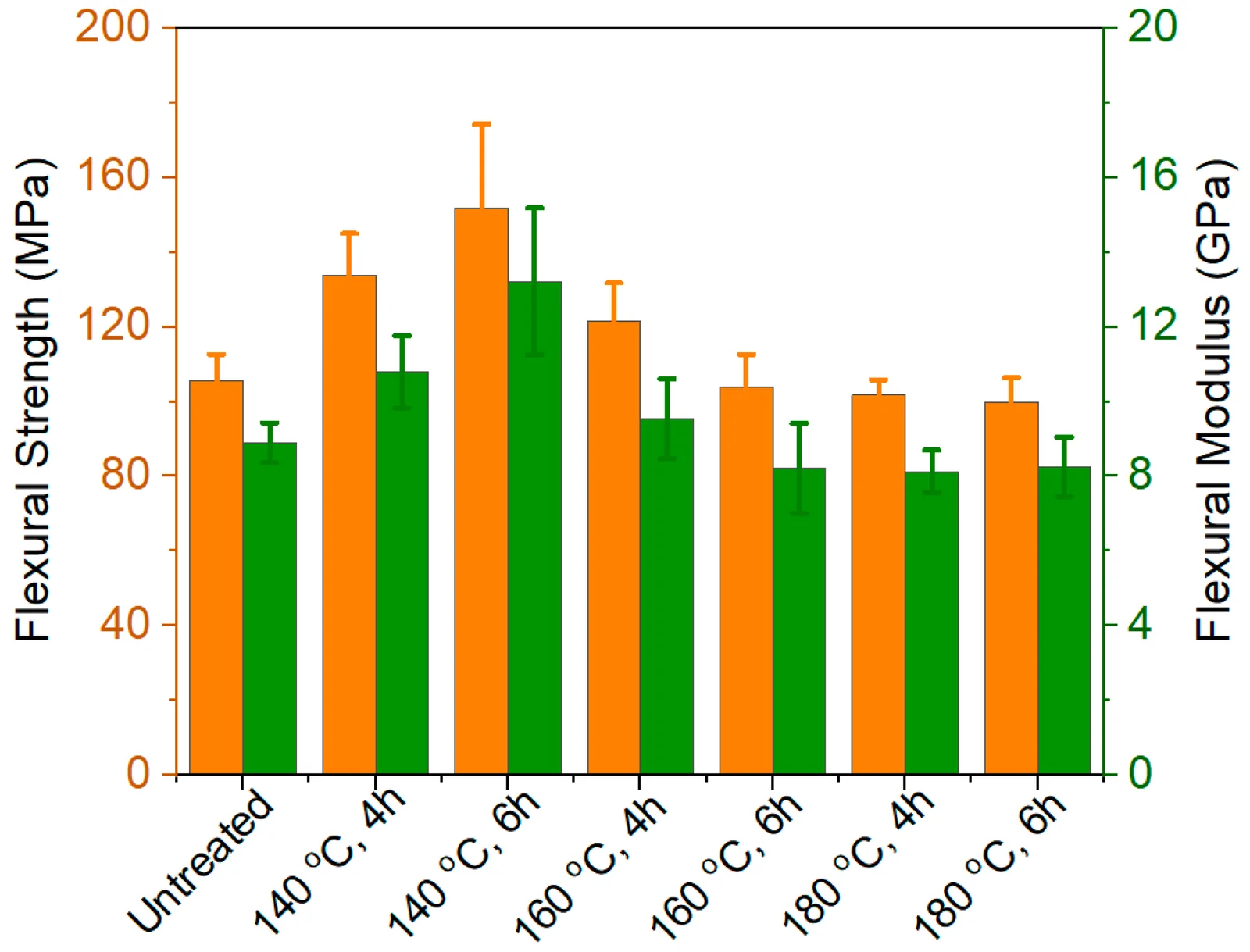
The flexural performance of the bamboo strips treated under different conditions.

Temperature and holding duration during heat treatment induce thermal degradation of polysaccharide components such as hemicellulose and cellulose inside the bamboo strips, while promoting condensation and crosslinking of lignin. These two effects jointly modify the microstructure of the bamboo strips and, thus, directly affect the flexural strength and flexural modulus of the material [24]. The test results of the flexural properties of the bamboo strips before and after heat treatment are shown in Figure 6.

The variation trends of flexural strength and flexural modulus of the bamboo strips before and after heat treatment were consistent, both increasing first and then decreasing, with peak values achieved at 140 °C for 6 h, as illustrated in Figure 6. During heat treatment at 140 °C, lignin reaches its glass transition temperature and undergoes melting rearrangement, filling the internal voids of cell walls to realize densification. Meanwhile, mild degradation of a small amount of hemicellulose occurs while the cellulose skeleton remains intact. The densified cell walls, together with reinforced-fiber interfaces, synergistically boost the flexural strength and flexural modulus of the bamboo strips. For samples treated at 140 °C for 6 h, the flexural strength and modulus rise by 43.72% and 48.54%, respectively. The effect of extending the holding time from 4 to 6 h was temperature-dependent. At 140 °C, the longer holding time further improved the flexural properties, whereas at 160 and 180 °C, prolonged exposure promoted thermal degradation and resulted in decreased flexural performance.

With a fixed holding duration, elevating the heat treatment temperature from 140 °C to 160 °C and 180 °C aggravates the thermal degradation of the hemicellulose and cellulose and causes excessive crosslinking and embrittlement of the lignin, which continuously reduces the flexural strength and modulus. All of the bamboo strips treated at a high temperature of 180 °C exhibit an inferior flexural performance compared with the untreated blank samples. In particular, for specimens treated at 180 °C for 6 h, the flexural strength and modulus decrease by 5.59% and 7.19%, respectively, compared with the untreated bamboo strips, indicating that high-temperature and long-duration heat treatment severely deteriorates the flexural properties of the bamboo strips [30,31].

A comparison of the tensile test results reveals that the flexural properties are far more sensitive to hemicellulose degradation and high-temperature embrittlement than the uniaxial tensile properties. This is because flexural loading generates both tension and compression zones within the material. Cavity collapse and interfacial crack propagation damage the structures of compressive and tensile regions simultaneously, so moderate and high-temperature heat treatments exert a more prominent adverse effect on the flexural performance of the bamboo strips.

Appropriate heat treatment can increase the density of the bamboo strips and simultaneously improve their mechanical properties. Excessive heat treatment, however, may reduce the density and cause synchronous deterioration of the mechanical performance. A color difference can serve as an intuitive visual indicator to predict the mechanical performance. Once the color difference exceeds the critical threshold, irreversible degradation occurs in the mechanical properties of the bamboo strips. To further evaluate the mechanical performance achieved by the present vacuum heat-treatment strategy, the results were compared with representative thermal-modification studies reported in the literature (Table S1). Compared with these previous treatments, the present work exhibits relatively pronounced improvements in tensile strength (+89.23%), flexural strength (+43.72%), and flexural modulus (+48.54%).

### 3.5. Effects of Heat Treatment on the Thermal Degradation Activation Energy and Thermal Stability of the Bamboo Strips

To investigate the effect of different heat treatment processes on the thermal degradation behavior of the bamboo strips, the Flynn–Wall–Ozawa (FWO) isoconversional method was adopted to calculate the thermal degradation activation energy (*E_a_*) of each sample within the pyrolysis conversion range of 10–50% based on TGA test data. Combined with the characteristic temperature of 5% mass loss (*T*_5%_), the influences of heat treatment temperature and holding duration on the thermal stability of the bamboo strips were analyzed.

#### 3.5.1. Effects of Heat Treatment on the Activation Energy of the Bamboo Strips

The activation energy data are summarized in Table 2. The thermal degradation activation energy of all bamboo strips shows a distinct upward trend with increasing pyrolysis conversion. For the untreated bamboo strips, the activation energy rises from 22.64 kJ/mol at a conversion of 10% to 30.16 kJ/mol at a conversion of 50%, and all heat-treated samples exhibit the same variation tendency.

At low-conversion stages, thermal degradation of the bamboo strips is dominated by the cleavage and decomposition of amorphous hemicellulose side chains, hydroxyl groups and small-molecule extracts, corresponding to a low reaction energy barrier. As pyrolysis proceeds, thermally labile components that decompose readily are gradually consumed, and the thermal degradation shifts toward densely packed cellulose crystalline regions and aromatic skeletons of lignin. Much higher energy is required to break hydrogen bonds and aromatic covalent bonds, which accounts for the continuous increase in activation energy with rising conversion [32,33].

Prolonging the holding time at 160 °C enables intensive degradation of hemicellulose and enriches cellulose and lignin, thereby increasing the pyrolysis activation energy and improving the thermal stability of the bamboo strips. The results reveal that the pyrolysis of the bamboo strips is a multi-component staged reaction, and the differences in activation energy among various components form an energy barrier gradient. The enhanced thermal stability is not a homogeneous reinforcement effect; instead, it arises from the dominant role of high heat-resistant skeleton structures after the consumption of thermally labile components.

lnβ = −(1.052·Ea)/(R·Tα) + C (where, β is the heating rate, Ea is the activation energy (kJ/mol), *Tα* is the absolute temperature at conversion α, and α is the fixed conversion degree).

**Figure 7:**
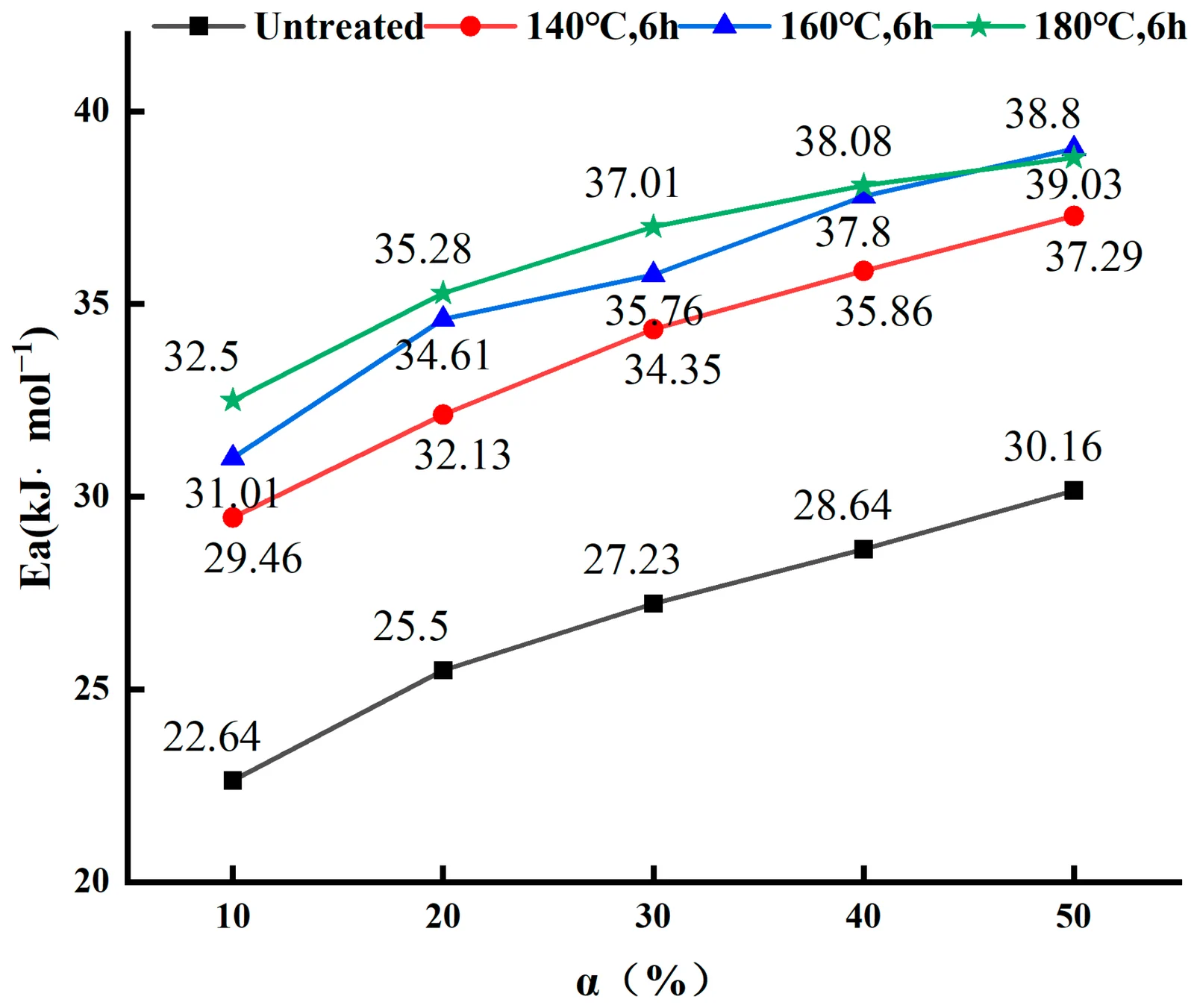
Variation in thermal degradation activation energy of bamboo with the pyrolysis-conversion rate under different heat treatments.

In the medium- and low-conversion range (α = 10–40%), the activation energy follows the general rule of thermal modification for lignocellulosic materials: the increase in heat treatment temperature gradually removes thermally labile components inside bamboo strips and optimizes the cell wall structure, which further improves the thermal stability of the material. At the deep-pyrolysis stage, with a conversion of 50%, the activation energy of the bamboo strips treated at 180 °C for 6 h is lower than that of samples treated at 160 °C for 6 h, as shown in Figure 7. This phenomenon originates from the adverse microstructural effects induced by excessive high-temperature long-duration heat treatment.

Treatment at 160 °C for 6 h represents a moderate modification condition. Under this condition, the bamboo strip structure remains intact with a small amount of highly stable hemicellulose fragments retained, resulting in a higher pyrolysis energy barrier. In contrast, treatment at 180 °C for 6 h causes damage to cellulose microcrystallites and local carbonization, accompanied by complete degradation of hemicellulose, which ultimately leads to a decrease in apparent activation energy [34,35,36].

#### 3.5.2. Effects of Heat Treatment Processes on the Thermal Stability of the Bamboo Strips

**Figure 8:**
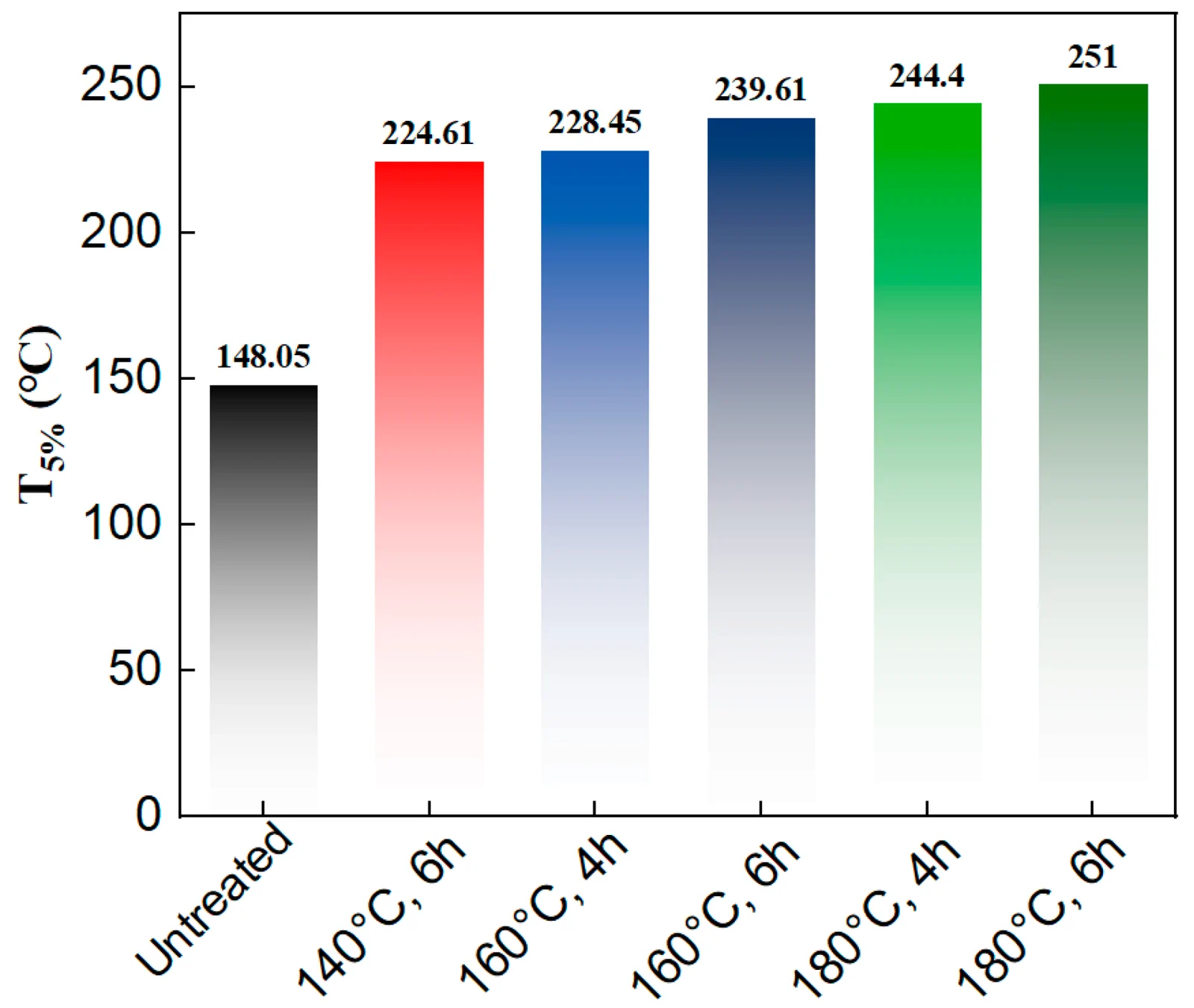
Comparison of the 5% mass loss temperature (T_5%_), reflecting the initial thermal stability of the bamboo under different heat treatments.

All heat-treated bamboo strips exhibited higher *T*_5%_ and higher *E_α_* across the full conversion range than the untreated controls (Figure 8), confirming that heat treatment effectively improved thermal stability. At a fixed holding duration, *T*_5%_ generally increased with the treatment temperature (180 > 160 > 140 °C). Extending the holding time from 4 h to 6 h at the same temperature further removed residual thermally labile components, producing minor additional increases in both *T*_5%_ and *E_α_*.

The mechanisms underlying these improvements differed by temperature level. Treatment at 140 °C removed only free water and a small fraction of unstable hemicellulose side chains, resulting in limited matrix reorganization and a marginal thermal stability gain. At 160 °C, substantial hemicellulose degradation occurred, accompanied by dehydration and ordering of amorphous cellulose regions and intermolecular lignin crosslinking; these changes produced a notable overall enhancement in thermal stability. At 180 °C, the hemicellulose was almost completely removed, cellulose crystallinity increased further, and the lignin underwent extensive polycondensation and crosslinking, yielding highly densified cell walls. These samples showed the highest *T*_5%_ and *E_α_* at low-to-medium conversion rates, indicating superior initial thermal resistance [20,26].

## 4. Conclusions

This study demonstrates that vacuum heat treatment modulates the properties of the bamboo strips through a temperature-driven competition between two opposing mechanisms, namely, densification via cell-wall shrinkage and degradation via thermal decomposition of hemicellulose and lignin, whose balance dictates the final density, color, microstructure, mechanical performance, and thermal stability. At 140 °C, the densification effect dominates, yielding maximum density, while temperatures of 160–180 °C progressively shift the balance toward degradation, triggering mass loss, microcrack formation, and consequential property deterioration. Color evolution, governed more by temperature than by holding time, follows a systematic chromatic transition that reflects the underlying chemical transformations of lignin and hemicellulose, with lightness and yellowness decreasing monotonically and redness exhibiting a non-monotonic response as treatment severity intensifies. Mechanical properties show a clear temperature-dependent optimum, with flexural performance peaking at 140 °C and tensile strength reaching its maximum at 160 °C for 4 h, beyond which the fibrous skeleton is irreversibly compromised, underscoring the trade-off between thermal modification and structural integrity. In terms of thermal resistance, the removal of labile hemicellulose elevates both the onset degradation temperature and pyrolysis activation energy, with the treatment at 160 °C for 6 h offering the best overall stability, though excessive treatment at 180 °C disrupts cellulose crystallites and reduces the activation energy at high conversion rates. Collectively, these findings indicate that vacuum heat treatment at 140–160 °C for approximately 4 h provides a favorable balance between the structural integrity, mechanical performance, and thermal stability of bamboo. This processing window can serve as a basis for selecting bamboo pretreatment conditions prior to the fabrication of bamboo–polymer composites.

## Figures and Tables

**Table 1:** Color difference variations of the bamboo strips before and after heat treatment.

Heat Treatment Conditions	L*	a*	b*	Δ*E*
Untreated	71.60	7.23	27.44	—
140 °C, 4 h	58.32 ± 1.31	15.00 ± 1.07	36.51 ± 0.86	17.86 ± 1.82
140 °C, 6 h	54.45 ± 1.34	15.66 ± 1.44	35.72 ± 2.08	20.95 ± 0.66
160 °C, 4 h	44.68 ± 0.77	16.54 ± 0.41	28.33 ± 1.42	28.52 ± 0.57
160 °C, 6 h	40.73 ± 1.62	15.96 ± 0.68	25.68 ± 1.49	32.16 ± 1.61
180 °C, 4 h	33.62 ± 1.31	13.73 ± 1.37	18.69 ± 1.01	39.53 ± 1.40
180 °C, 6 h	30.31 ± 0.28	12.24 ± 0.96	15.40 ± 0.60	43.32 ± 0.21

— indicates not applicable because the untreated bamboo was used as the reference for calculating ΔE.

**Table 2:** Thermal degradation activation energy of bamboo with different heat treatments at various pyrolysis conversion rates.

α (%)	Untreated	140 °C, 6 h	160 °C, 4 h	160 °C, 6 h	180 °C, 4 h	180 °C, 6 h
10	22.64	29.46	30.74	31.01	31.56	32.5
20	25.5	32.13	33.12	34.61	34.18	35.28
30	27.23	34.35	35.57	35.76	35.93	37.01
40	28.64	35.86	37.18	37.8	37.34	38.08
50	30.16	37.29	38.24	39.03	38.18	38.8

## Data Availability

The original contributions presented in this study are included in the article. Further inquiries can be directed to the corresponding authors.

## Supplementary Materials

The following supporting information can be downloaded at: https://www.mdpi.com/article/10.3390/polym18172098/s1, References [37,38] are cited in the supplementary materials, Figure S1. SEM images of bamboo samples subjected to vacuum heat treatment at different temperatures: (a) 140 °C for 6 h; (b) 160 °C for 6 h; (c) 180 °C for 6 h. Table S1. Comparison of mechanical-property changes in thermally modified bamboo reported in previous studies. Table S2. Two-way ANOVA of the effects of treatment temperature and holding time on ΔE.
