# Supplementary material for: Effects of Heat Treatment on the Mechanical Properties and Thermal Stability of Bamboo
Source: Polymers (Basel). 2026 Aug 29;18(17):2098. doi: 10.3390/polym18172098 (PMC13568039; doi:10.3390/polym18172098)
Supplement: Supplementary file 1 [file polymers-18-02098-s001.zip › polymers-4491559-supplementary.pdf]

Supporting Information

Effects of Heat Treatment on the Mechanical Properties and Thermal Stability of Bamboo

Zilu Liang *, Haiyun Jiang, and Yimin Tan *

School of Packaging and Materials Engineering, Hunan University of Technology, Zhuzhou 412007, China
* Correspondence: lzl98qindao@126.com (Z.L.); csfutanyimin@126.com (Y.T.)

Supplementary Figures S1

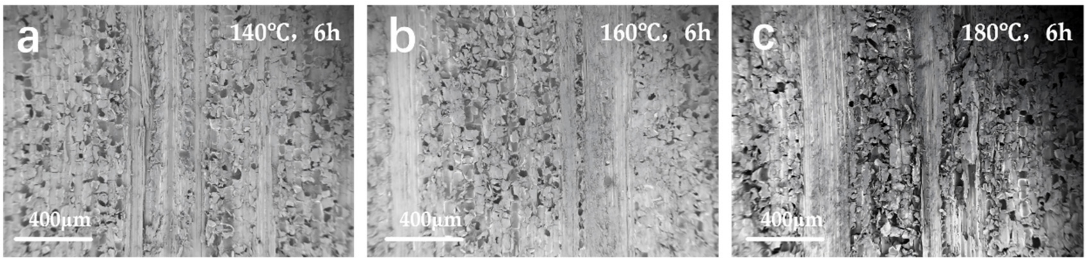

Figure S1. SEM images of bamboo samples subjected to vacuum heat treatment at different temperatures: (a) 140 °C for 6 h; (b) 160 °C for 6 h; (c) 180 °C for 6 h.

Supplementary Table S1

Table S1. Comparison of mechanical-property changes in thermally modified bamboo reported in previous studies

Study	Treatment	Key mechanical improvement
Wang et al. (2020)[1]	Saturated steam, 140 °C/30 min	MOR +10.8%; MOE +17.3%
Li et al. (2023)[2]	Pressurized heat treatment, 130 °C/1 h	Bending strength +3.5%; longitudinal compression +10.6%
Li et al. (2022)[3]	Hydrothermal, 120–160 °C	Severe treatment caused MOR/MOE reductions; no pronounced simultaneous enhancement
Present work	Vacuum heat treatment, 140–160 °C	Tensile strength +89.23%; flexural strength +43.72%; flexural modulus +48.54%

Table S2. Two-way ANOVA of the effects of treatment temperature and holding time on ΔE.

Source of variation	Sum of squares	df	Mean square	F value	p value	Contribution (%)
Temperature	1454.221	2	727.111	503.81	<0.001	95.23
Holding time	55.193	1	55.193	38.24	<0.001	3.61
Temperature × holding time	0.399	2	0.199	0.138	0.872	0.03
Residual	17.319	12	1.443	—	—	1.13
Total	1527.131	17	—	—	—	100.00

References

1. Wang, X., et al., *Effect of high-temperature saturated steam treatment on the physical, chemical, and mechanical properties of moso bamboo.* Journal of Wood Science, 2020. **66**(1): p. 52.
2. Li, D., et al., *Effects of Heat Treatment under Different Pressures on the Properties of Bamboo.* Polymers, 2023. **15**(14): p. 3074.
3. Li, X., et al., *Effect of High-Temperature Hydrothermal Treatment on Chemical, Mechanical, Physical, and Surface Properties of Moso Bamboo.* Forests, 2022. **13**(5): p. 712.
